# Bibliography

**Published:** 1905-01

**Authors:** 


### Bibliography.

ORTHODONTIA AND ORTHOPÆDIA OF THE FACE. By Victor Hugo Jackson, M.D., D.D.S., Member of the National Dental Association; Professor of Orthodontia in the Dental Department of the University of Buffalo; Fellow of the New York Academy of Medicine; Member of the American Medical Association, etc. With seven hundred and sixty original illustrations. J. B. Lippincott Company, Philadelphia and London, 1904.

The dental profession has for several years been very familiar with the author's work in orthodontia, through his articles in the dental periodicals, and have come to regard him as an authority in this particular line of dental operations. It is, therefore, with unusual satisfaction that he has concentrated his long experience in book form to be readily accessible to all workers in this direction, and this includes, in degree, the entire dental profession.

There is no branch of dental work that has attracted more attention than that of the regulation of malposed teeth. The books devoted to this subject, both in America and Europe, attest the importance of this branch of dental procedures. This should have been expected, for the disfigurement of the human face through irregular dentures has demanded the highest attainment in skill in its treatment. The earlier efforts in this direction lacked system, and the development of methods was of very slow growth; and, further, the philosophic discussion of the subject was not, to any extent, embraced in the earlier teachings. The works of Farrar, Talbot, Angle, Guilford, and others in this country have broadened the subject until it has become very attractive for the thoughtful and inventive mind, for each new case presents difficulties to surmount and interest.

Dr. Jackson's work is eminently practical. He does not enter into wide discussion of the probable etiological factors that lead to irregular development, but is mainly satisfied with a comparatively brief allusion to these, covering some fifty-four pages. The fact that the teeth must be returned to normal positions and the lines of beauty restored, seems to be the paramount idea in the author's mind.

Dr. Jackson is known as having developed a system. It cannot be said he originated the idea of springs, for wires and springs have been used more or less satisfactorily since the period when it was first attempted to regulate teeth. While this is true, these efforts were but a crude application of force, as a rule, and were very often more productive of injury than of benefit to the patient. Dr. Jackson's system has, however, moulded these earlier efforts into procedures that give to all the appliances originality, and exhibit the active and earnest study of an inventive mind.

The author says of his system, "For many years I have applied in my own practice the principles explained in this book, and the results warrant me in giving a detailed description of my system. In the mean time I have been urging upon the profession, in writings and public demonstrations, the use of the spring as a force in regulating, and it is especially gratifying to note its more general adoption in place of screw-pressure.

"Evolution is the law of our science as of other studies. To realize this, one has but to compare the methods of a few years ago with those of to-day. As it stands, my system of correcting irregularities includes important modifications of the appliances originally presented by me. The changes are in the line of simplicity and practicability."

The care taken by the author in the study of cases is evidenced by the following quotation as to his method of procedure in each case. He says, "The first thing that strikes us favorably in a face is exactness of proportion, then harmony of the features. When a case is presented for the regulation of the teeth, it is important that the contour of the features be studied before the mouth is examined or models are made. Careful note should be taken of the apparent changes required; these should accord with the type and temperament, whether well nourished or developed; the profile, including the size of the nose and the orifices, the width between the alæ nasi, the prominence of the cheek-bones, and the fulness or recession in the region of the upper lip. The relative position of the chin to the forehead should be noted to determine where the deformity is situated, and whether the upper or lower arch should be made more or less prominent. The features should be studied both in repose and when animated, especially when laughing."

These are valuable suggestions to the beginner in this work, the neglect of which has oftentimes produced deformity in attempted regulation. In the reviewer's opinion, upon this will mainly depend whether the operator should spread the jaw, or attempt other means to overcome a contracted arch. Whether extraction should ever be resorted to in regulating must largely depend upon the conclusion arrived at after the study, advised by the author, has been completed.

The difficulty in removing plaster impressions is overcome by the author through forcing water under the lip with the force of a syringe. He regards this "as an invaluable procedure."

Under the head of "Anchorages and Appliances" the author sums up the advantage of his system:

"First. The simplicity and ease of construction of the apparatus.

"Second. It is equally applicable for the regulation of the teeth of the child or the adult.

"Third. It is suitable for all forms of irregularity, doing away with the use of a plate in a large majority of cases.

"Fourth. It is well retained in apposition with the teeth, and when the appliance is properly made it causes no inconvenience. It does not interfere materially with speech or with the occlusion of the teeth, even when an apparatus is used in both the upper and the lower arch at the same time.

"Fifth. While the anchorage is sufficiently firm for all practicable purposes, the appliance can usually be removed by the patient, thus favoring cleanliness.

"Sixth. It can be so arranged that some of the teeth to be moved later in the process of regulating can be employed to assist the anchorage, thereby lessening the disturbance of the anchorage-teeth when extreme force is applied."

He further contends that the force is well under control, and

"the appliance can be made of almost any of the precious metals that are springy or base metals." The "attachment base wire and spring wire" are simple and quickly prepared. Almost any of the appliances can be continued in use as retainers.

On page 134 the author shows a decided antagonism to the jack-screw, for he says, "I have not found the jack-screw of especial value in any case, and owing to its interference with the tongue in pronunciation and mastication, suggest that its use, both for pushing and pulling the teeth, be discontinued in favor of methods more agreeable to the patient." While he thus objects to its use, and recognizing "that some operators employ it," he gives illustrations of its employment in several special cases. He states with emphasis his objections to the screw or intermittent pressure for regulating purposes as advocated by Dr. Farrar in what he terms his "positive system." The author says, "The law of nature is, that alternating pressure and relief cause hypertrophy, increased growth, and hardening, while constant pressure causes atrophy or absorption." While this may be true of alternate periods of irritation and rest, it does not seem to the reviewer to apply to the Farrar system. Theoretically the intermittent pressure should produce a hardening of the tissue, but in no sense is this pressure delayed to a period injurious, for the benefit of short periods of partial rest seems of special value to the overstrained nervous system of the patient, while the constant pressure may produce serious lesions in careless hands. This has so repeatedly been observed by the reviewer, that while he is not devoted to any one method of moving teeth, yet he must object to the inculcation of the idea that "the tissues involved in regulating are tolerant of any kind of direct moderate force, whether constant or intermittent." The author seems to contradict this positive assertion in another part of the book, for he there states: "The too rapid movement of the teeth is always objectionable. The alveolar process is gradually absorbed as the roots of the teeth are moved toward one another, and if the apices of the roots are moved too rapidly through the process there is always danger of strangulation of the pulps." This latter is the ever-present danger in inexperienced hands.

The face-masks, illustrated by the author, beginning on page 242, with insets, are of special interest, representing, as they do, the original deformity with the appearance after correction. These give a better idea of the defect, with the improvement, than a photograph of the face could possibly present.

On page 253 (Fig. 281) is a very interesting illustration of an original design by the author to break up the habit of thumb-sucking. It consists of a thumb-cot with a small serrated leather strip attached to the cot, and this again attached to a bracelet at the wrist, held fast by a small padlock, the key of which was kept by the mother. It broke up the habit in a few days.

"General Orthopædia of the Face," including "Nasal Deformities," might, as a chapter in a book on regulating, be profitably omitted. It belongs more properly to the work of the surgeon, and will not, in all probability, come within the province of the orthodontist.

The book as a whole must impress the practical man as one well adapted to his needs. It covers, in its cases, all that the operator will ever have fall into his hands for regulating. The author has spent but little time in theorizing as to the cause of these abnormal conditions, but recognizes the importance of this in his chapter on "Etiology." His desire has been apparently to show in the most direct and simple manner how the irregularities may be remedied by his system, and the reader must acknowledge that his application of force reduces the regulation of teeth to very simple and inexpensive appliances.

It is thought that this book will have a wide circulation; indeed, its original character will compel every practitioner to make it part of his library for frequent reference. It is impossible to have too many of these practical works. They will eventually fuse, as it were, into each other, and in time we will have, not a series of systems, as is the case at present, but one system that can be taught successfully to the coming generations of orthodontists.

The students at our colleges will need this book to broaden their knowledge in a practical direction. There is too much dependence upon certain methods at the present time. Teaching, to be of value, must have elasticity and be prepared to meet all difficulties as they arise without an entire dependence on any one system or any one theory.

The book is prepared by the J. B. Lippincott Company, in type, paper, etc., in their usual excellent style of work. Some of the illustrations are not altogether satisfactory from either a dental or artistic view, but these are probably old cuts used by the author to illustrate his papers. The more recent illustrations are well prepared.

The Physician's Visiting List for 1905. Fifty-fourth year of its publication. P. Blakiston's Son & Co., Philadelphia.

This more than a half-century old publication is offered again for the use of physicians for 1905.

It is doubtful whether any other of similar character can show as long a period of usefulness. For the medical practitioner, for whom it is specially arranged, there would seem that there could be no better arrangement for daily memoranda.

